# Characterization of a Novel, Cold-Adapted, and Thermostable Laccase-Like Enzyme With High Tolerance for Organic Solvents and Salt and Potent Dye Decolorization Ability, Derived From a Marine Metagenomic Library

**DOI:** 10.3389/fmicb.2018.02998

**Published:** 2018-12-05

**Authors:** Qihao Yang, Mengle Zhang, Manman Zhang, Chunqing Wang, Yanyan Liu, Xinjiong Fan, He Li

**Affiliations:** ^1^School of Basic Courses, Guangdong Pharmaceutical University, Guangzhou, China; ^2^School of Basic Medical Sciences, Anhui Medical University, Hefei, China

**Keywords:** laccase, thermostability, organic solvent tolerance, halotolerance, dye decolorization

## Abstract

Synthetic dyes are widely used in many industries, but they cause serious environmental problems due to their carcinogenic and mutagenic properties. In contrast to traditional physical and chemical treatments, biodegradation is generally considered an environmental-friendly, efficient, and inexpensive way to eliminate dye contaminants. Here, a novel laccase-like enzyme Lac1326 was cloned from a marine metagenomic library. It showed a maximum activity at 60°C, and it retained more than 40% of its maximal activity at 10°C and more than 50% at 20–70°C. Interestingly, the laccase behaved stably below 50°C, even in commonly used water-miscible organic solvents. The enzyme decolorized all tested dyes with high decolorization efficiency. This thermostable enzyme with high decolorization activity and excellent tolerance of organic solvents and salt has remarkable potential for bioremediation of dye wastewater. It is thus proposed as an industrial enzyme.

## Introduction

Nowadays, more than 100,000 different dye structures are synthesized, with more than 0.7 million tons of dyestuff produced annually in diverse industries like chemical, pharmaceutical, biomedical, food, leather, textile, plastic, and printing industries (Yamjala et al., 2016; Paz et al., 2017). Most of these dyes are poisonous or mutagenic to numerous living organisms (Elshafei et al., 2015, 2017). More than 11% of applied dyes are lost during the dying process, and this contaminated wastewater must be treated before discharging it into the environment (Alhassani et al., 2007). Common physical and chemical processes to treat these effluents include adsorption, precipitation, coagulation, flocculation, and filtration. However, these processes can be ineffective and costly and sometimes generate hazardous byproducts (Holkar et al., 2014; Paz et al., 2017). Therefore, the development of an enzymatic method that acts on a wide range of dyes is in great demand. An enzymatic process has several advantages over the classical physical and chemical methods, including low energy costs, ease of control, and ecofriendly impact (Othman et al., 2018).

Laccases (benzenediol: oxygen oxidoreductase, EC 1.10.3.2) are multi-copper enzymes that catalyze the mono-electronic oxidation of substrates in the presence of molecular oxygen as an electron acceptor (Ghodake et al., 2018). Because they can oxidize both phenolic and non-phenolic compounds in the presence or absence of a mediator, laccases have been considered for use in dye decolorization (Tavares et al., 2009). According to the literature, many laccases have been isolated from different microorganisms, including fungi and bacteria, and some show high decolorization capacity (Michniewicz et al., 2006; Fang et al., 2012; Liu et al., 2017; Othman et al., 2018). However, biological treatment of dye wastewater usually involves environments with high salinity, numerous organic solvents, and high temperatures. Most laccases lose activity under these extreme conditions (Santhanam et al., 2011). It is therefore important to identify new laccases with special properties, such as high tolerance for salt, organic solvents, and temperature.

Marine environments have a rich diversity of microorganisms and natural resources (Kennedy et al., 2011). They also have extreme variations in pressure, salinity, temperature, and nutrients (Kennedy et al., 2008; Theerachat et al., 2018). Local microbes have adapted habitat-related characteristics, such as salt and pH tolerance, psychrotolerance, and thermostability, making their enzymes attractive for bioprocesses (Theerachat et al., 2018). In this study, we constructed a metagenomic library from marine sediment samples from the South China Sea. We then cloned a novel laccase gene *lac1326*, using the activity-based functional method. The recombinant enzyme Lac1326 is expressed in *Escherichia coli* BL21 (DE3) in soluble form. It showed high activity in a wide temperature range and had excellent tolerance to temperature, organic solvents, and salt. We also investigated the potential use of the laccase for dye decolorization.

## Materials and Methods

### Strains, Vector, and Chemicals

*Escherichia coli* DH5α (Invitrogen, Carlsbad, CA, United States) was used as the cloning host, *E. coli* BL21 (DE3) served as the expression host (Novagen, Madison, WI, United States), and pET-32a (+) (Novagen) was used for protein expression. All restriction endonucleases and ligases were purchased from TaKaRa (Dalian, China). All other chemicals and reagents were from Sigma-Aldrich (St. Louis, MO, United States) or Sangon (Shanghai, China) unless indicated otherwise.

### DNA Extraction, Metagenomic Library Construction, and Laccase Gene Screening

The marine sediment samples were collected from the Qiongdongnan basin in the South China Sea (17°33′17′′N, 110°30∘′26′′E) at a depth of 716.8 m. Samples were stored at -80°C until the DNA extraction was performed. The total genomic DNA from the marine sediment sample was extracted using a QIAamp DNA Tool Mini Kit according to the recommendations of product manual (QIAGEN, Hilden, Germany). Genomic DNA was partially digested with *Bam*HI.

DNA fragments (2–10 kb) were purified using a QIAquick Gel Extraction Kit (QIAGEN) and inserted into the pUC118 vector (TaKaRa), which had been previously digested with *Bam*HI and dephosphorylated with calf intestine alkaline phosphatase. Next, the recombinant plasmid was transformed into *E. coli* DH5α and cultured on LB agar plates containing IPTG (isopropyl β-D-1-thiogalactopyranoside; 24 μg/mL), 5-bromo-4-chloro-3-indolyl-β-D-galactopyranoside (40 μg/mL), and ampicillin (100 μg/mL) at 37°C overnight.

The white colonies on the plate were transferred into 10-mL test tubes containing 5 mL of LB medium supplemented with ampicillin (100 μg/mL) and 1 mM of IPTG at 37°C overnight. A 2-mL culture was harvested by centrifugation and lysed with 200 μL of lysis reagent containing 50 mM of Tris–HCl, 1% Triton X-100, and lysozyme (1 mg/mL) at pH 7.5. ABTS was added to the final concentration of 1 mM. A putative ABTS-oxidizing screening was performed by visualizing the green color that resulted from hydrolysis of ABTS. Finally, the DNA of positive colonies was sequenced on the ABI 377 DNA sequencer and analyzed using ORF-finder and BlastX from the National Center for Biotechnology Information^1^.

### Cloning and Overexpression of the Laccase Gene in *E. coli* and Purification of the Recombinant Protein

The putative laccase activity gene was amplified from the pUC118-lac plasmid by using the primers’ introduced *Sac*I and *Hin*dIII restriction sites for cloning to pET32a. The PCR primers for *lac1326* amplification were *lac1326*-f: (5′-CGC GAGCTC ATG CTC GCA TTC GAT TTT TTC CTC-3′) and *lac1326*-r (5′-AGC AAGCTT CAA TGG CGC GTC TGG GAC CCA GGT-3′). The underlined sequences represent the recognition sites of restriction enzymes *Sac*I and *Hin*dIII, respectively. The PCR products were digested using *Sac*I and *Hin*dIII and then ligated to expression vector pET-32a (+), which was treated with the same restriction endonuclease. The recombinant vectors were transformed into *E. coli* BL21 (DE3), and the cells were plated on LB agar containing ampicillin (100 μg/mL).

The transformants were grown in a 100-mL flask containing 10 mL of LB medium (100 μg/mL concentration of ampicillin) at 37°C until OD_600_ reached 1.2. Next, induction was initiated by adding IPTG to the final concentration of 1.0 mM, and the cultures were incubated at 30°C for 16 h with shaking at 150 rpm. Cells were then collected by centrifugation (8,000 *g* for 10 min at 4°C). Purification of the recombinant proteins was performed using a HisBind Purification Kit (Novagen) according to the product manual. Finally, the purified enzyme was collected and stored at -20°C for further characterization.

### Determination of Molecular Mass

The molecular mass of the denatured protein was determined by sodium dodecyl sulfate-polyacrylamide gel electrophoresis (SDS-PAGE). The native-PAGE (Non-denaturing PAGE) was conducted at 25°C using 15% polyacrylamide gel. The molecular mass of the enzyme subunit was estimated using protein markers (TaKaRa) as standards. Proteins were stained with Coomassie brilliant blue G-250.

### Enzyme Assay

Laccase activity was measured at 60°C using ABTS (2,2′-Azino-bis[3-ethylbenzothiazoline-6-sulfonate]) as the substrate. Oxidation of ABTS was detected at 420 nm (ε_420__nm_ = 36,000 M^-1^ cm^-1^), as in Martins et al. (2002). The reaction mixture contained 0.04 M of Britton-Robinson buffer (pH 7.0). The reaction was started by adding ABTS to a final concentration of 1 mM. One unit was defined as the amount of enzyme that oxidized 1 μmol of substrate per minute. All assays were performed in triplicate.

### Determination of Substrate Specificity and Kinetic Parameters

Several typical substrates of laccases were used to investigate the substrate specificity of the recombinant enzyme. These substrates were ABTS (ε_420__nm_ = 36,000 M^-1^ cm^-1^), guaiacol (ε_465__nm_ = 12,000 M^-1^ cm^-1^), 2,6-dimethoxyphenol (2,6-DMP, ε_468__nm_ = 14,800 M^-1^ cm^-1^), 1,2-dihydroxybenzene (catechol, ε_450__nm_ = 2,211 M^-1^ cm^-1^), and syringaldazine (ε_330__nm_ = 65,000 M^-1^ cm^-1^), respectively (Martins et al., 2002; Lu et al., 2012). The chemical structures of the tested substrates were shown in Supplementary Figure S1. The enzymatic activity was tested under the standard conditions. The kinetic parameters (*K*_m_ and *k*_cat_) for the recombinant enzyme were determined by analyzing enzymatic activity in 0.04 M of Britton-Robinson buffer (pH 7.0) at 60°C using different substrates. Kinetic data were fitted to hyperbola by using the Michaelis–Menten equation. Kinetic analyses by curve fitting were performed with the Origin software (OriginLab Corporation, Northampton, MA, United States). Finally, the substrate order was confirmed by comparing catalytic efficiencies (*k*_cat_/*K*_m_) of the enzyme toward the abovementioned substrates.

### Effect of Temperature and pH on Activity and Stability of the Recombinant Lac1326

The optimum temperature of the recombinant Lac1326 was determined by testing laccase activity at pH 7.0 in temperatures between 0 and 75°C, using ABTS as the substrate. Thermostability was evaluated by pre-incubation of the purified Lac1326 for 2 h in 0.04 M of Britton-Robinson buffer (pH 7.0) at 0–75°C. Then, the residual enzyme activity was tested. The optimum pH was tested using ABTS as a substrate at 60°C in Britton-Robinson buffer (pH 4–10). The pH stability was evaluated after incubation of Lac1326 for 4 h at 25°C in the previously mentioned solution.

### Effect of Organic Solvents and High Salt on Activity of the Recombinant Lac1326

The effects of various chemicals on Lac1326 activity were investigated by preincubating the enzyme in various concentrations of the organic solvents (methanol, ethanol, acetone, acetonitrile, and dimethyl sulfoxide) for 12 h at 25°C. The residual activity then was measured, as previously described, and expressed as a percentage of activity in the absence of the added compound. The maximum value was set as 100%, where 100% was a concentration of 22.82 U/mg. Additionally, with ABTS as the substrate, the effect was studied by measuring the residual enzyme activity under standard assay conditions, after preincubating the enzyme in different concentrations of NaCl (100, 500, and 1000 mM) at 25°C.

### Dye Decolorization

Six dyes (amaranth, Coomassie brilliant blue, bromophenol blue, acid violet 7, Congo red, and indigo carmine) were used to evaluate the decolorization ability of Lac1326. The chemical structures of the tested dyes were shown in Supplementary Figure S2. The decolorization rate of the dyes was tested in the presence or absence of ABTS as mediator (Kumar et al., 2018). The reaction mixtures contained the dyes (50 mg/L), 1 mM of ABTS, 0.04 M of Britton-Robinson buffer (pH 7.0), and 10 μg of purified laccase (22.82 U/mg). The reaction mixtures were incubated at 25°C and shaken at 150 rpm for 12 h. Decolorization ability was determined spectrophotometrically as the relative decrease of absorbance. The test wavelengths for each were as follows: amaranth, 520 nm; Coomassie brilliant blue, 465 nm; bromophenol blue, 593 nm; acid violet 7, 524 nm; Congo red, 488 nm; and indigo carmine, 610 nm. All reactions were performed in triplicate.

### Nucleotide Sequence Accession Number

The nucleotide sequence data reported here has been deposited in the nucleotide sequence databases (GenBank) under accession number KP752045.

## Results and Discussion

### Screening for Laccase Using the Marine Metagenomic Library

The classical approach to screen for microorganisms with a specific trait of interest requires culturing. A metagenomics approach, which does not require culturing, has been successfully applied to exploit novel enzymes (Handelsman, 2004; Fan et al., 2017). In this study, a metagenomic library consisting of approximately 60,000 clones was successfully constructed using the total DNA extracted from marine sediment samples from the South China Sea. The average insert size of each clone was about 5.2 kb. Thus, the metagenomic library represents about 312 MB of community DNA from soil microbes. One putative ABTS-oxidizing clone was visually identified by a green appearance. One *E. coli* DH5α clone harboring the pUC118-lac showed ABTS-oxidizing activity.

### Genetic Characterization

**FIGURE 1:**
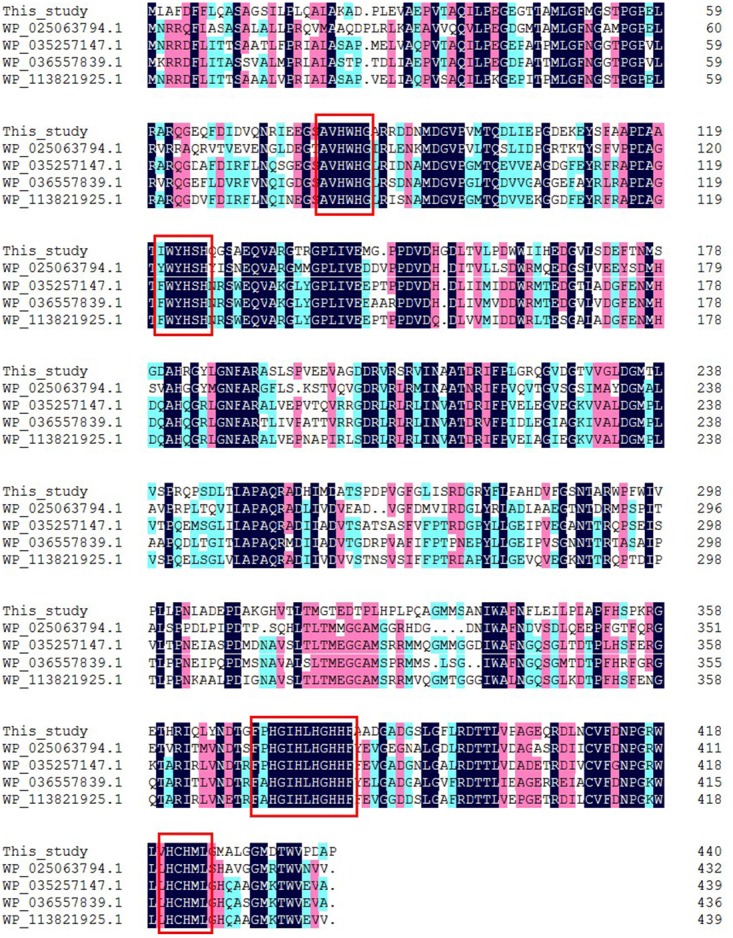
Amino acid sequence alignment of Lac1326 and its homologs. The conserved amino acid residues of active sites are shown with a box.

The complete insert DNA sequence of pUC118-lac was determined. Sequence data from this clone revealed the presence of one open reading frame of 1326 bp, encoding a 441-amino-acid protein with a predicted molecular mass of 47.94 kDa. The putative laccase gene was designated *lac1326*. A protein BLAST (BLASTp) search in the NCBI databases revealed that *lac1326* is a member of the multicopper oxidase protein family. It shared the highest identity of 64% with the multicopper oxidase of *Oceanibulbus indolifex*, as well as 61% identity with a laccase (Lac15) from a marine microbial metagenome and 60% identity with a laccase from uncultured bacterium. These results suggest that *lac1326* was probably a novel oxidase from uncultured marine bacteria. Furthermore, multiple sequence alignments of *lac1326* and four other homologous multicopper oxidases and laccases allowed the identification of four conserved copper-binding domains of *lac1326*, which are characteristic for laccases (Figure 1). The results showed that *lac1326* was similar to these multicopper oxidases and laccases in four conserved copper-binding domains.

### Overexpression and Purification of Recombinant Lac1326

The gene *lac1326* was expressed in *E. coli* BL21 (DE3). The cell lysate was clear, suggesting that no inclusion bodies had formed. The crude recombinant protein was further purified by Ni-NTA chromatography, and the result suggested that Ni-NTA chromatography of the cell lysate led to 5.75-fold purification and 78.38% of activity yield (Table 1). Specific activity of the purified enzyme was 22.82 U/mg.

**Table 1:** Purification of the recombinant Lac1326.

Purification step	Total protein (mg)	Total activity (U)	Specific activity (U/mg)	Fold purification	Activity yield (%)
Cell lysate	52.64	208.98	3.97	1.00	100.00
Ni-NTA chromatography	8.28	163.80	22.82	5.75	78.38

**FIGURE 2:**
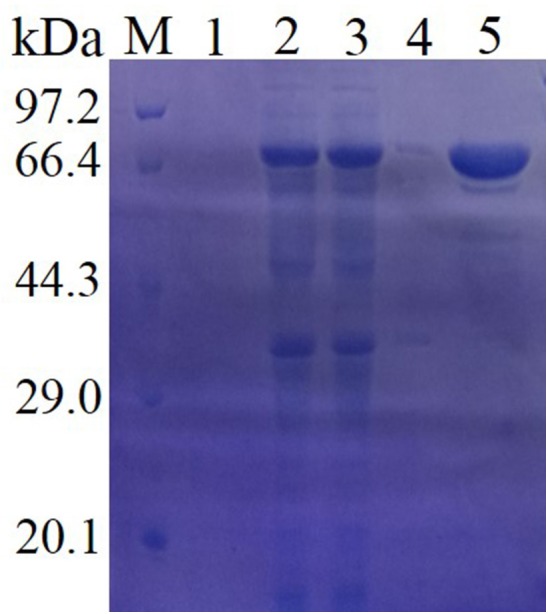
Sodium dodecyl sulfate-polyacrylamide gel electrophoresis analysis of the purified recombinant Lac1326. Protein markers (lane Maker) stained with Coomassie brilliant blue, centrifugal supernatant of fermentation broth (lane 1), cell lysates (lane 2), supernatant of cell lysates (lane 3), centrifugal sedimentation of cell lysates (lane 4), and purified target protein (lane 5).

Then, the purified enzyme and the crude enzyme (the supernatant from the cell lysates) were applied to SDS-PAGE (Figure 2) and native SDS-PAGE (Supplementary Figure S3) together to determine the molecular mass of the recombinant enzyme. The native-PAGE result was shown in Supplementary Figure S3. The protein moved as single band on native-PAGE, which demonstrated that Lac1326 was a monomeric enzyme. SDS-PAGE analysis demonstrated that the purified enzyme migrated as a single band, with an approximate molecular mass of 66 kDa, in accordance with its predicted molecular mass (47.94 kDa) plus the N-terminal fusion protein of the expression vector (about 18 kDa). The highest expression level of *lac1326* in *E. coli* was a concentration of about 227 mg/L when the cell was induced at 30°C for 16 h. In Figure 2, the target protein was mainly found in the supernatant of cell lysates, while there were a few in centrifugal supernatant of fermentation broth (lane 1) and centrifugal sedimentation of cell lysates (lane 3). The results showed that the produced laccase was intracellular, not bounded to the cells or secreted outside the cell.

### Kinetic Parameters and Substrate Specificity of Recombinant Lac1326

Laccase could oxidize several substrates, and produce different colors (Supplementary Figure S4). Table 2 shows the kinetic parameters of Lac1326 for several substrates. Generally, catalytic efficiency (*k*_cat_/*K*_m_) is considered an indicator of enzymatic specificity. Due to its highest *k*_cat_/*K*_m_ value, ABTS was regarded as the best substrate for Lac1326, followed by syringaldazine, Catechol, 2,6-DMP, and guaiacol. The *K*_m_ of Lac1326 was different from other laccases. Kumari et al. (2018) reported the *K*_m_ for ABTS was 2.4 μM × 10^3^ μM at 60°C, 3.8 μM × 10^3^ μM at 55°C, and 4.4 μM × 10^3^ μM at 50°C; the *K*_m_ for SGZ was 15.3 μM at 40°C, and 16.7 μM at 35°C, for *Thermus thermophilus* laccase TthLAC (Kumari et al., 2018). Values of *K*_m_ of extracellular laccases from *Pleurotus ostreatus* were 230–14,000 μM for 2,6-DMP, 70–370 μM for ABTS, 1200–3100 μM for guaiacol. *K*_m_ values of BOX from *P. ostreatus* was lower than the corresponding values determined for other laccases isolated from the same source (Pakhadnia et al., 2009). The laccase from *Trametes pubescens* is characterized by *K*_m_ = 14 μM for ABTS, *K*_m_ of laccase from *Thelephora terrestris* is 3 μM for SGZ (Kanunfre and Zancan, 1998; Galhaup et al., 2002). Low *K*_m_ value demonstrated that Lac1326 shows positive affinity toward ABTS. High activity is another attractive property of enzymes for practical applications. For comparison, for laccase TthLAC, its *k*_cat_ was 4.8 s^-1^ for ABTS at 60°C and it was 10.3 s^-1^ for SGZ at 40°C (Kumari et al., 2018). The *k*_cat_ of Lac1326 was higher than that of TthLAC, but lower than that of *Bacillus pumilus* laccase (Reiss et al., 2011). The catalytic activity would be improved by directed mutagenesis in further study.

**Table 2:** Kinetic parameters and substrate specificity of the recombinant Lac1326.

Substrates	*K*_m_	*k*_cat_	k_cat_/*K*_m_	Specific
	(μM)	(S^-1^)	(S^-1^μM^-1^)	activity (U/mg)
2,6-DMP	654	9.43	0.014	1.12
ABTS	210	34.39	0.16	22.82
Guaiacol	4900	1.34	0.00027	0.64
Catechol	507	13.99	0.028	10.61
Syringaldazine	831	24.13	0.029	17.16

From the results of genetic characterization and substrate specificity of recombinant Lac1326, it’s speculated that lac1326 was probably a novel laccase-like from uncultured marine bacteria.

### Effect of pH and Temperature on the Activity and Stability of Recombinant Lac1326

**FIGURE 3:**
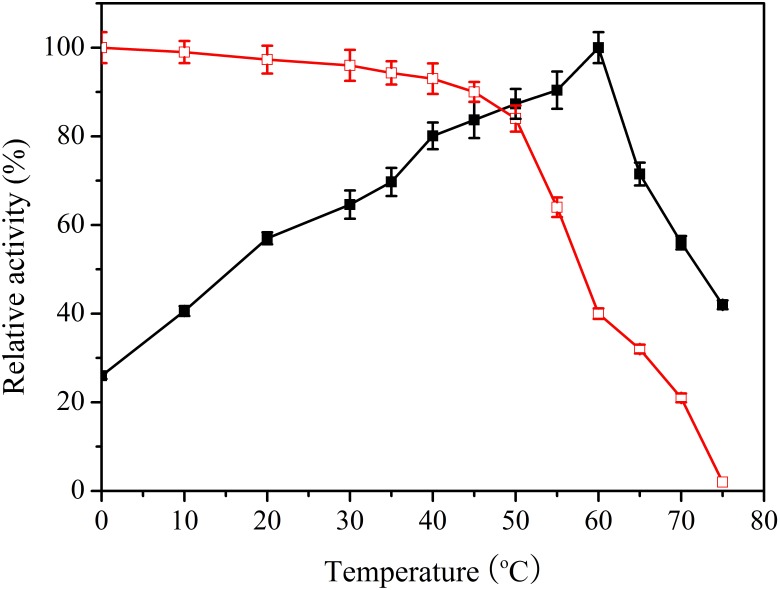
Effect of temperature on activity 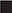 and stability (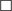) of the recombinant Lac1326. The purified enzyme was preincubated at 0–75°C for 2 h. Data points are the average of triplicate measurements, and error bars represent the standard deviation. Specific activity of the purified enzyme was 22.82 U/mg at optimal conditions, and the maximum value was set as 100%.

To measure the optimal temperature of Lac1326, catalytic activity was estimated at different temperatures ranging from 0 to 75°C, with ABTS as a substrate. Specific activity of the purified enzyme was 22.82 U/mg at optimal conditions, and the maximum value was set as 100%. Maximum activity occurred at 60°C, and 26.5% of maximum activity was retained at 0°C and more than 40% at 10°C (Figure 3). This remarkable activity at cold temperatures indicates that Lac1326 could be a cold-adapted protein. Moreover, the enzyme displayed more than 50% of its maximal activity at 20–70°C, indicating that this laccase possessed good adaptability in a wide temperature range. Lac1326 is likely endowed with marine-specific characteristic (i.e., cold adaptation), because the extreme marine environment can modulate enzyme characteristics (Duarte et al., 2018).

Despite the relevance of these laccases for industrial and environmental applications, studies related to cold-adapted proteins are scarce. To analyze thermostability of Lac1326, the proteins were pre-treated at different temperatures. Figure 3 shows the residual activities of the laccases. According to the literature, most cold-adapted enzymes exhibit poor thermal stability at temperatures above 40°C, and their activity decreases as temperature increases (Ke et al., 2018). Interestingly, the laccase behaved stably at 0–50°C (the relative activity exceeded 80% of maximum). Even at 70°C, after 2 h of incubation, more than 20% of the initial laccase activity remained, suggesting that this cold-adapted laccase possessed good thermostability. Additionally, Lac1326 showed different enzymatic properties compared to laccases from other marine organisms. For example, two laccases from *Cerrena unicolor* 137 (isolated from a terrestrial habitat) are unstable at high temperatures: Lacc I loses 100% of its activity after 20 min, and Lacc II loses 90% of its activity after 60 min at 70°C (Michniewicz et al., 2006; D’Souza-Ticlo et al., 2009).

**FIGURE 4:**
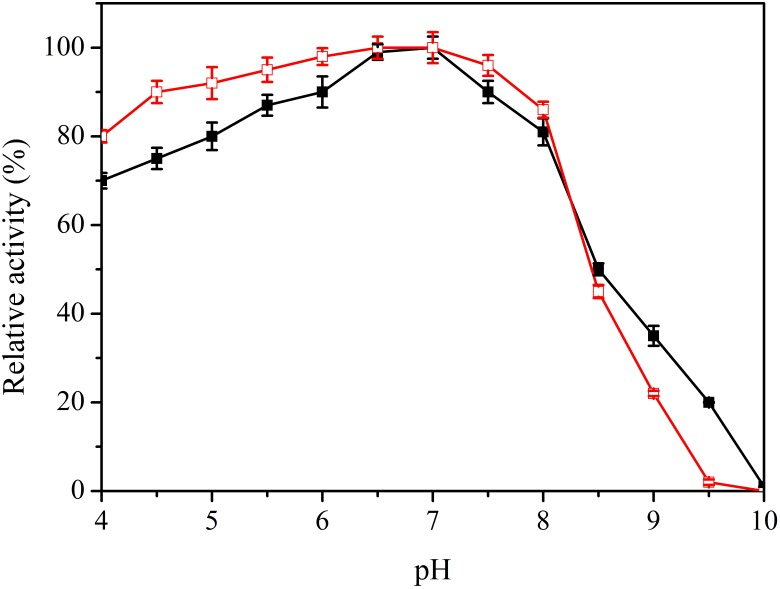
Effect of pH on activity 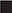 and stability (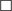) of the recombinant Lac1326. The purified enzyme was preincubated in different buffers for 4 h at 25°C. Data points are the average of triplicate measurements, and error bars represent the standard deviation. Specific activity of the purified enzyme was 22.82 U/mg at optimal conditions, and the maximum value was set as 100%.

The pH value is an important factor in laccase catalysis. Specific activity of the purified enzyme was 22.82 U/mg at optimal conditions, and the maximum value was set as 100%. Lac1326 displayed an optimal pH of 7.0 toward ABTS (Figure 4) and showed high activity in the pH range 5.0–8.0 (relative activity more than 80%). Regarding pH stability, it was observed that the laccase retained more than 80% of its activity in the pH range 4.0–8.0 (Figure 4). The pH of most industrial wastewater, such as that from the textile industry, is neutral to alkaline (Manu and Chaudhari, 2002; Jahmeerbacus et al., 2004), thus indicating the usefulness of Lac1326 in wastewater treatment.

Other laccases from marine organisms do not show similar characteristics. For example, the marine bacterial laccase Lac21 is highly stable at pH 6.0–8.0 but unstable at low pH values (Fang et al., 2012). Lac 15, isolated from the same habitat, shows an optimum pH toward syringaldazine of 7.5 and stability at pH 5.5–9.0 (Fang et al., 2011). The optimum pH with ABTS as the substrate for the Lac IId from the marine-adapted fungus *C. unicolor* MTCC 5159 was found at 3 (Michniewicz et al., 2006).

### Effect of Organic Solvents and High Salt on Activity of the Recombinant Lac1326

Biological treatment of black liquor and textile dye wastewater usually requires enzymes, which can maintain their activities in environments of high organic solvents and salinity (Qiao et al., 2017). Thus, it is necessary to investigate the effect of organic solvents and high salt on activity and stability of Lac1326. We found that the purified laccase was stable in some commonly used water-miscible organic solvents. Enzyme activity was significantly increased in 10% of all tested organic solvents (Table 3). More than 80% of enzyme activity remained in 30% of the tested solvents.

**Table 3:** Effect of organic solvents and high salt on activity of the recombinant Lac1326.

Organic solvents	Concentration	Residual activity (%)
None	0 (%, v/v)	100
Methanol	10 (%, v/v)	158.5 ± 3.2
	30 (%, v/v)	115.2 ± 2.7
	50 (%, v/v)	109.6 ± 2.8
Acetone	10 (%, v/v)	237.2 ± 4.7
	30 (%, v/v)	189.2 ± 2.9
	50 (%, v/v)	135.6 ± 3.8
Ethanol	10 (%, v/v)	170.9 ± 3.5
	30 (%, v/v)	106.4 ± 2.1
	50 (%, v/v)	89.2 ± 2.5
Acetonitrile	10 (%, v/v)	103.7 ± 2.2
	30 (%, v/v)	82.2 ± 1.8
	50 (%, v/v)	39.7 ± 2.5
Dimethyl sulphoxide	10 (%, v/v)	116.4 ± 2.8
	30 (%, v/v)	87.1 ± 2.9
	50 (%, v/v)	73.8 ± 2.2
NaCl	100 mM	204.6 ± 4.2
	500 mM	167.2 ± 3.8
	1000 mM	104.3 ± 2.9

The recombinant laccase was extremely resistant toward methanol and acetone, and enzyme activities increased in the 50% concentrations. The result suggested that Lac1326 was resistant to these organic solvents. The same behavior was observed with CotA-laccase from *Bacillus subtilis* cjp3 (Qiao et al., 2017). Fungal laccases of *Trametes versicolor* and *Pleurotus ostreatus* were rapidly inactivated in solutions with more than 10% of the organic solvents (Liu et al., 2009).

The negative effects of organic solvents may be due to enzymatic conformational changes, loss of water from protein structures, and solvent penetration into the active sites (Rodakiewicz-Nowak and Jarosz-Wilkolazka, 2007). Interestingly, Lac1326 also showed good halotolerance and significantly enhanced activity, maintaining 204.6% and 167.2% of its original activity in the presence of 100 mM and 500 mM of NaCl, respectively. The results indicate that Lac1326 might be suitable for kraft pulp biobleaching and wastewater decolorization, because chlorine is used at high concentrations in those industries.

Two new bacterial laccases from the South China Sea metagenomics library, lac15 and lac21, show high Cl^-^ tolerance (Fang et al., 2011, 2012). Because organic solvents and high salt concentrations are usually found in wastewater (Ogugbue et al., 2011; Qiao et al., 2017), the excellent organic solvency and salt tolerance of Lac1326 has potential for industrial dye effluent treatment.

### Dye Decolorization

Laccases degrade many environmental pollutants, including textile dyes, personal-care products, and micropollutants (Kadam et al., 2017). In this study, six dyes (amaranth, Coomassie brilliant blue, bromophenol blue, acid violet 7, Congo red, and indigo carmine) were selected for determining the decolorization efficacy of laccase Lac1326. As shown in Table 4, the decolorization amounts of the tested dyes were less than 50% in the absence of ABTS. Even worse, amaranth and acid violet 7 showed no decolorization during the incubation time.

**Table 4:** Decolorization rate of Lac1326 for several dyes in the absence/presence of the redox mediator.

	Decolorization rate (%)	Decolorization rate (%)
Dyes	without ABTS	with ABTS
Amaranth	<5	75.7 ± 1.8
Coomassie brilliant blue	15 ± 1.1	95 ± 1.9
Bromophenol blue	21 ± 1.7	80 ± 3.6
Acid violet 7	<5	77.6 ± 1.7
Congo red	32 ± 2.8	95.4 ± 3.2
Indigo carmine	45 ± 2.5	100 ± 0

The use of mediators can promote degradation of pollutants. Mediators improve the catalytic efficiency of laccases by enhancing electron transfer (Galletti et al., 2014). When using ABTS as a mediator, the laccase Lac1326 efficiently decolorized all tested dyes. Indigo carmine was fully degraded, and the decolorization amounts of other tested dyes exceeded 75%. Decolorization tests showed that the recombinant Lac1326 achieved higher efficiency in decolorization of synthetic dyes when in the presence of ABTS as a mediator, perhaps because it produced cation radicals with the laccase. Cation radicals can take electrons from other electron-rich compounds, such as synthetic dyes, and then return to their original states (Lu et al., 2012).

Lac1326 performed similarly to previous findings for the CotA-laccase. Qiao et al. (2017) found that CotA-laccase decolorized all tested dyes in the presence of acetosyringone as a mediator; however, it could not decolorize the dyes without the mediator under the same conditions. Our results were opposite to those of a previous study showing that Lac3T93 and its parent enzyme effectively degrades many dyes in the absence of a redox mediator (Liu et al., 2011). Moreover, in this study, the decolorization temperature was 25°C (room temperature), not the optimal 60°C temperature of Lac1326, because most textile industry wastewater is treated at room temperature. We speculate that the dye decolorization efficiency of Lac1326 will increase substantially at increased temperatures (40–60°C). Therefore, the high dye decolorization ability of Lac1326 at room temperature makes it a good candidate for use in textile dye decolorization and bioremediation of textile wastewater.

## Conclusion

Due to their broad substrate and catalyzed reaction spectrums, laccases are industrially relevant enzymes for decolorizing recalcitrant dyes. In this study, a novel laccase-like gene, lac1326, was cloned from a marine metagenomic library using an activity-based functional method. Lac1326 was successfully expressed in *E. coli*. The recombinant enzyme was purified and characterized. It showed high activity in a wide temperature range, good thermostability, and excellent tolerance of organic solvents and salt. In the presence of ABTS as a mediator, the enzyme efficiently decolorized all tested dyes. These properties indicate its potential usefulness in many industrial applications for treating dye wastewater.

## Author Contributions

QY have done enzyme assay and determined substrate specificity and kinetic parameters. MeZ have determined the effect of temperature and pH on activity and stability of the recombinant enzyme. MaZ have determined the effect of organic solvents and high salt on activity of the recombinant enzyme. CW have done the SDS-PAGE and native-PAGE. YL have tested dye decolorization of the enzyme. XF have cloned the laccase gene, and revised the manuscript. HL have written the manuscript, conceived the study and supervised the experiments. All authors have read and approved the manuscript.

## Conflict of Interest Statement

The authors declare that the research was conducted in the absence of any commercial or financial relationships that could be construed as a potential conflict of interest.

## Footnotes

1 http://www.ncbi.nlm.nih.gov

## Supplementary Material

FIGURE S1 The chemical structures of the tested substrates.

FIGURE S2 The chemical structures of the tested dyes.

FIGURE S3 Native-PAGE of the purified recombinant Lac1326. Protein markers (lane Maker) stained with Coomassie brilliant blue, and native Lac1326 stained with Coomassie brilliant blue (lane 1), native Lac1326 that catalyzed the oxidation of syringaldazine (activity staining) (lane 2).

FIGURE S4 The color reaction of the purified recombinant Lac1326 against several substrates.

## References

[B1] Alhassani H. A. Rauf M. A. Ashraf S. S. (2007). Efficient microbial degradation of Toluidine Blue dye by *Brevibacillus* sp. *Dyes Pigm.* 75 395–400. 10.1016/j.dyepig.2006.06.019

[B2] D’Souza-Ticlo D. Sharma D. Raghukumar C. (2009). A thermostable metal-tolerant laccase with bioremediation potential from a marine-derived fungus. *Mar. Biotechnol.* 11 725–737. 10.1007/s10126-009-9187-0 19283431

[B3] Duarte A. W. F. dos Santos J. A. Vianna M. V. Vieira J. M. F. Mallagutti V. H. Inforsato F. J. (2018). Cold-adapted enzymes produced by fungi from terrestrial and marine Antarctic environments. *Crit. Rev. Biotechnol.* 38 600–619. 10.1080/07388551.2017.1379468 29228814

[B4] Elshafei A. Elsayed M. Hassan M. Haroun B. Othman A. Farrag A. (2017). Biodecolorization of six synthetic dyes by *Pleurotus ostreatus* ARC280 laccase in presence and absence of hydroxybenzotriazole (HBT). *Annu. Res. Rev. Biol.* 15 1–16. 10.9734/ARRB/2017/35644

[B5] Elshafei A. Othman A. Hassan M. Haroun B. Elsayed M. Farrag A. (2015). Catalyzed mediator-based decolorization of five synthetic dyes by Pleurotus ostreatus ARC280 laccase. *Biotechnol. J.* 9 1–15. 10.9734/BBJ/2015/19505

[B6] Fan X. J. Liang M. J. Wang L. Chen R. Li H. Liu X. L. (2017). Aii810, a novel cold-adapted N-Acylhomoserine lactonase discovered in a metagenome, can strongly attenuate *Pseudomonas aeruginosa* virulence factors and biofilm formation. *Front. Microbiol.* 8:1950. 10.3389/fmicb.2017.01950 29067011 PMC5641347

[B7] Fang Z. M. Li T. L. Chang F. Zhou P. Fang W. Hong Y. Z. (2012). A new marine bacterial laccase with chloride-enhancing, alkaline-dependent activity and dye decolorization ability. *Bioresour. Technol.* 111 36–41. 10.1016/j.biortech.2012.01.172 22377476

[B8] Fang Z. M. Li T. L. Wang Q. Zhang X. C. Peng H. Fang W. (2011). A bacterial laccase from marine microbial metagenome exhibiting chloride tolerance and dye decolorization ability. *Appl. Microbiol. Biot.* 89 1103–1110. 10.1007/s00253-010-2934-3 20963410

[B9] Galhaup C. Goller S. Peterbauer C. Strauss J. Haltrich D. (2002). Characterization of the major laccase isoenzyme from *Trametes pubescens* and regulation of its synthesis by metal ions. *Microbiology* 148 2159–2169. 10.1099/00221287-148-7-2159 12101303

[B10] Galletti P. Pori M. Funiciello F. Soldati R. Ballardini A. Giacomini D. (2014). Laccase-mediator system for alcohol oxidation to carbonyls or carboxylic acids: toward a sustainable synthesis of profens. *ChemSusChem* 7 2684–2689. 10.1002/cssc.201402136 25044433

[B11] Ghodake G. S. Yang J. Shinde S. S. Mistry B. M. Kim D. Y. Sung J. S. (2018). Paper waste extracted α-cellulose fibers super-magnetized and chitosan functionalized for covalent laccase immobilization. *Bioresour. Technol.* 261 420–427. 10.1016/j.biortech.2018.04.051 29698891

[B12] Handelsman J. (2004). Metagenomics: application of genomics to uncultured microorganisms. *Microbiol. Mol. Biol. R.* 68 669–685. 10.1128/MMBR.68.4.669-685.2004 15590779 PMC539003

[B13] Holkar C. R. Pandit A. B. Pinjari D. V. (2014). Kinetics of biological decolorisation of anthraquinone based reactive Blue 19 using an isolated strain of *Enterobacter* sp. F NCIM 5545. *Bioresour. Technol.* 173 342–351. 10.1016/j.biortech.2014.09.108 25310871

[B14] Jahmeerbacus M. I. Kistamah N. Ramgulam R. B. (2004). Fuzzy control of dyebath pH in exhaust dyeing. *Coloration Technol.* 120 51–55. 10.1111/j.1478-4408.2004.tb00206.x

[B15] Kadam A. A. Jang J. Lee D. S. (2017). Supermagnetically tuned halloysite nanotubes functionalized with aminosilane for covalent laccase immobilization. *ACS Appl. Mater. Inter.* 9 15492–15501. 10.1021/acsami.7b02531 28418639

[B16] Kanunfre C. C. Zancan G. T. (1998). Physiology of exolaccase production by *Thelephora terrestris*. *FEMS Microbiol. Lett.* 161 151–156. 10.1111/j.1574-6968.1998.tb12942.x

[B17] Ke M. M. Ramesh B. Hang Y. A. Liu Z. D. (2018). Engineering and characterization of a novel low temperature active and thermo stable esterase from marine. *Enterobacter cloacae*. *Int. J. Biol. Macromol.* 118 304–310. 10.1016/j.ijbiomac.2018.05.193 29842953

[B18] Kennedy J. Marchesi J. R. Dobson A. D. W. (2008). Marine metagenomics: strategies for the discovery of novel enzymes with biotechnological applications from marine environments. *Microb. Cell Fact.* 7:27. 10.1186/1475-2859-7-27 18717988 PMC2538500

[B19] Kennedy J. O’Leary N. D. Kiran G. S. Morrissey J. P. O’Gara F. Selvin J. (2011). Functional metagenomic strategies for the discovery of novel enzymes and biosurfactants with biotechnological applications from marine ecosystems. *J. Appl. Microbiol.* 111 787–799. 10.1111/j.1365-2672.2011.05106.x 21777355

[B20] Kumar M. Mishra A. Singh S. S. Srivastava S. Thakur I. S. (2018). Expression and characterization of novel laccase gene from *Pandoraea* sp. ISTKB and its application. *Int. J. Biol. Macromol.* 115 308–316. 10.1016/j.ijbiomac.2018.04.079 29665388

[B21] Kumari A. Kishor N. Guptasarma P. (2018). Characterization of a mildly alkalophilic and thermostable recombinant *Thermus thermophilus* laccase with applications in decolourization of dyes. *Biotechnol. Lett.* 40 285–295. 10.1007/s10529-017-2461-8 29063287

[B22] Liu L. Lin Z. Zheng T. Lin L. Zheng C. Lin Z. (2009). Fermentation optimization and characterization of the laccase from *Pleurotus ostreatus* strain 10969. *Enzyme Microb. Technol.* 44 426–433. 10.1016/j.enzmictec.2009.02.008

[B23] Liu Y. H. Huang L. Guo W. Jia L. B. Fu Y. Gui S. (2017). Cloning, expression, and characterization of a thermostable and pH-stable laccase from *Klebsiella pneumoniae* and its application to dye decolorization. *Process Biochem.* 53 125–134. 10.1016/j.procbio.2016.11.015

[B24] Liu Y. H. Ye M. Lu Y. Zhang X. Li G. (2011). Improving the decolorization for textile dyes of a metagenome-derived alkaline laccase by directed evolution. *Appl. Microbiol. Biotechnol.* 91 667–675. 10.1007/s00253-011-3292-5 21523474

[B25] Lu L. Zhao M. Wang T. N. Zhao L. Y. Du M. H. Li T. L. (2012). Characterization and dye decolorization ability of an alkaline resistant and organic solvents tolerant laccase from Bacillus licheniformis LS04. *Bioresour. Technol.* 115 35–40. 10.1016/j.biortech.2011.07.111 21868217

[B26] Manu B. Chaudhari S. (2002). Anaerobic decolorisation of simulated textile wastewater containing azo dyes. *Bioresour. Technol.* 82 225–231. 10.1016/S0960-8524(01)00190-0 11991070

[B27] Martins L. O. Soares C. M. Pereira M. M. Teixeira M. Costa T. Jones G. H. (2002). Molecular and biochemical characterization of a highly stable bacterial laccase that occurs as a structural component of the Bacillus subtilis endospore coat. *J. Biol. Chem.* 277 18849–18859. 10.1074/jbc.M200827200 11884407

[B28] Michniewicz A. Ullrich R. Ledakowicz S. Hofrichter M. (2006). The white-rot fungus *Cerrena unicolor* strain 137 produces two laccase isoforms with different physico-chemical and catalytic properties. *Appl. Microbiol. Biot.* 69 682–688. 10.1007/s00253-005-0015-9 15983808

[B29] Ogugbue C. J. Sawidis T. Oranusi N. A. (2011). Evaluation of colour removal in synthetic saline wastewater containing azo dyes using an immobilized halotolerant cell system. *Ecol. Eng.* 37 2056–2060. 10.1016/j.ecoleng.2011.09.003

[B30] Othman A. M. Elsayed M. A. Elshafei A. M. Hassan M. M. (2018). Purification and biochemical characterization of two isolated laccase isoforms from *Agaricus bisporus* CU13 and their potency in dye decolorization. *Int. J. Biol. Macromol.* 113 1142–1148. 10.1016/j.ijbiomac.2018.03.043 29545062

[B31] Pakhadnia Y. G. Malinouski N. I. Lapko A. G. (2009). Purification and characteristics of an enzyme with both bilirubin oxidase and laccase activities from mycelium of the basidiomycete *Pleurotus ostreatus*. *Biochemistry* 74 1027–1034. 19916914 10.1134/s0006297909090119

[B32] Paz A. Carballo J. José Pérez M. Domínguez J. M. (2017). Biological treatment of model dyes and textile wastewaters. *Chemosphere* 181 168–177. 10.1016/j.chemosphere.2017.04.046 28437742

[B33] Qiao W. C. Chu J. P. Ding S. J. Song X. Yu L. (2017). Characterization of a thermo-alkali-stable laccase from *Bacillus subtilis* cjp3 and its application in dyes decolorization. *J. Environ. Sci. Heal. Part A* 52 710–717. 10.1080/10934529.2017.1301747 28358283

[B34] Reiss R. Ihssen J. Thöny-Meyer L. (2011). Bacillus pumilus laccase: a heat stable enzyme with a wide substrate spectrum. *BMC Biotechnol.* 11:9. 10.1186/1472-6750-11-9 21266052 PMC3041658

[B35] Rodakiewicz-Nowak J. Jarosz-Wilkolazka A. (2007). Catalytic activity of *Cerrena unicolor* laccase in aqueous solutions of water-miscible organic solvents–Experimental and numerical description. *J. Mol. Catal. B-enzym.* 44 53–59. 10.1016/j.molcatb.2006.08.005

[B36] Santhanam N. Vivanco J. M. Decker S. R. Reardon K. F. (2011). Expression of industrially relevant laccases: prokaryotic style. *Trends Biotechnol.* 29 480–489. 10.1016/j.tibtech.2011.04.005 21640417

[B37] Tavares A. P. M. Cristóvão R. O. Loureiro J. M. Boaventura R. A. R. Macedo E. A. (2009). Application of statistical experimental methodology to optimize reactive dye decolourization by commercial laccase. *J. Hazard. Mater.* 162 1255–1260. 10.1016/j.jhazmat.2008.06.014 18639377

[B38] Theerachat M. Guieysse D. Morel S. Remaud-Siméon M. Chulalaksananukul W. (2018). Laccases from marine organisms and their applications in the biodegradation of toxic and environmental pollutants: a review. *Appl. Biochem. Biotechnol.* 10.1007/s12010-018-2829-9 [Epub ahead of print] 30009326

[B39] Yamjala K. Nainar M. S. Ramisetti N. R. (2016). Methods for the analysis of azo dyes employed in food industry - a review. *Food Chem.* 192 813–824. 10.1016/j.foodchem.2015.07.085 26304415

