# Supplementary figures and images for: Characterization of a Novel, Cold-Adapted, and Thermostable Laccase-Like Enzyme With High Tolerance for Organic Solvents and Salt and Potent Dye Decolorization Ability, Derived From a Marine Metagenomic Library
Source: Front Microbiol. 2018 Dec 5;9:2998. doi: 10.3389/fmicb.2018.02998 (PMC6290062; doi:10.3389/fmicb.2018.02998)

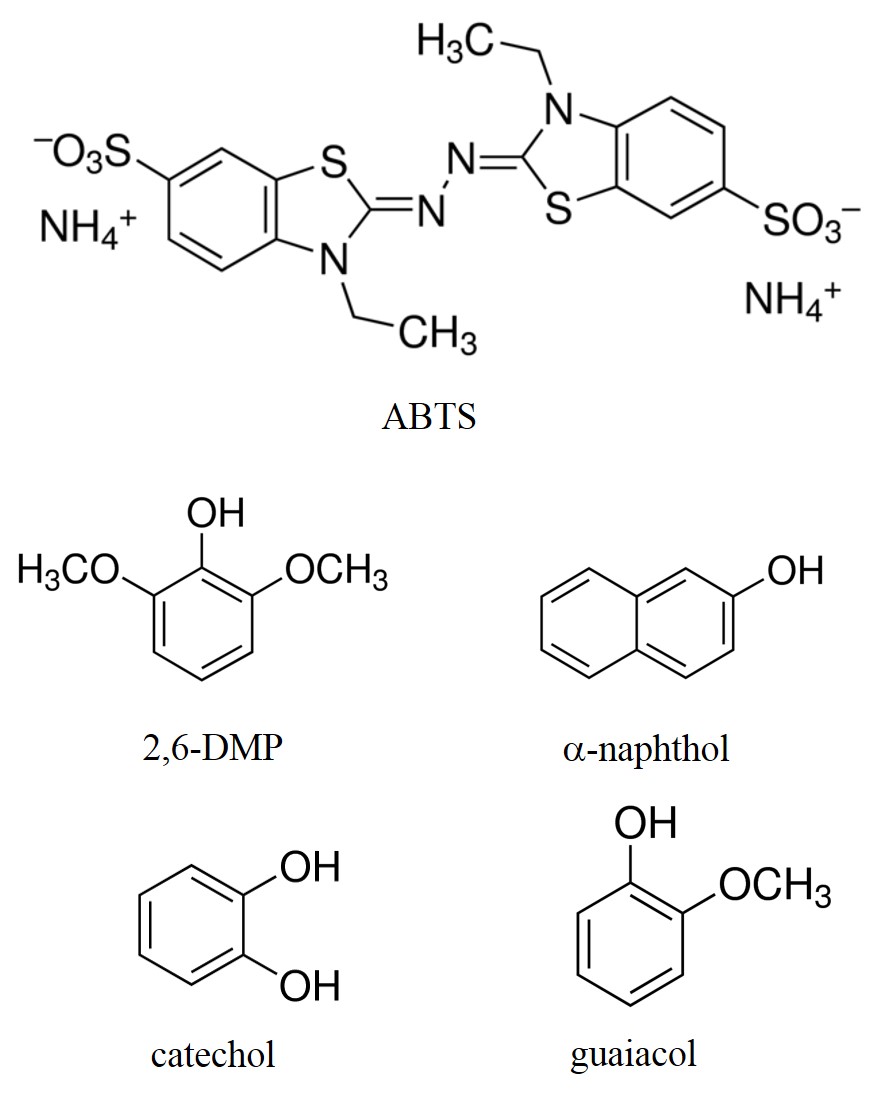

Supplement: FIGURE S1 — The chemical structures of the tested substrates. [file Image_1.JPEG]

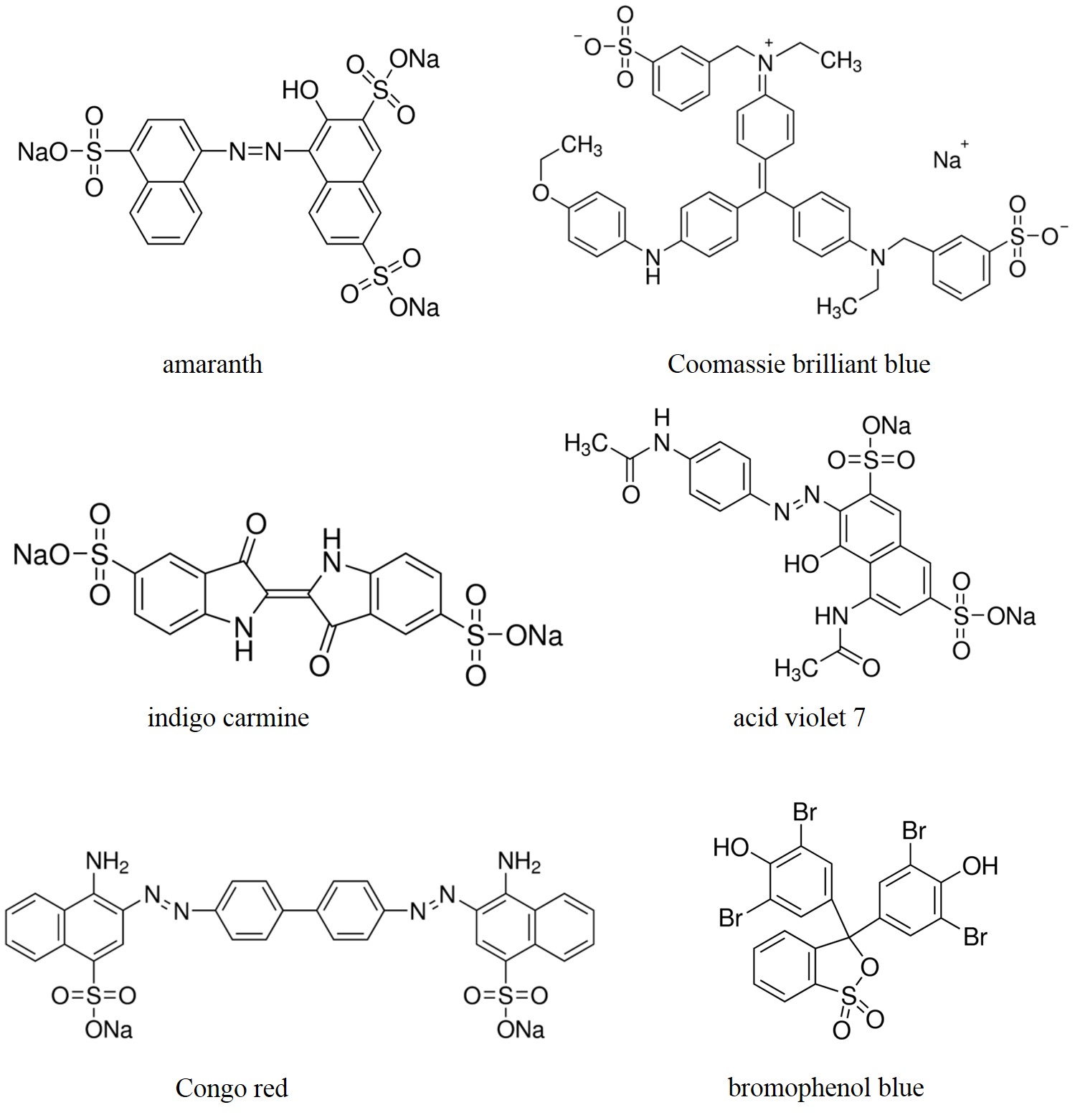

Supplement: FIGURE S2 — The chemical structures of the tested dyes. [file Image_2.JPEG]

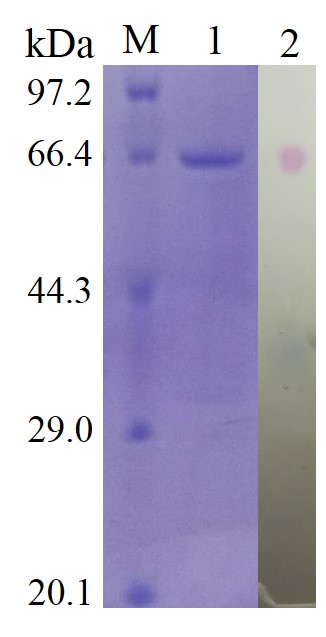

Supplement: FIGURE S3 — Native-PAGE of the purified recombinant Lac1326. Protein markers (lane Maker) stained with Coomassie brilliant blue, and native Lac1326 stained with Coomassie brilliant blue (lane 1), native Lac1326 that catalyzed the oxidation of syringaldazine (activity staining) (lane 2). [file Image_3.JPEG]

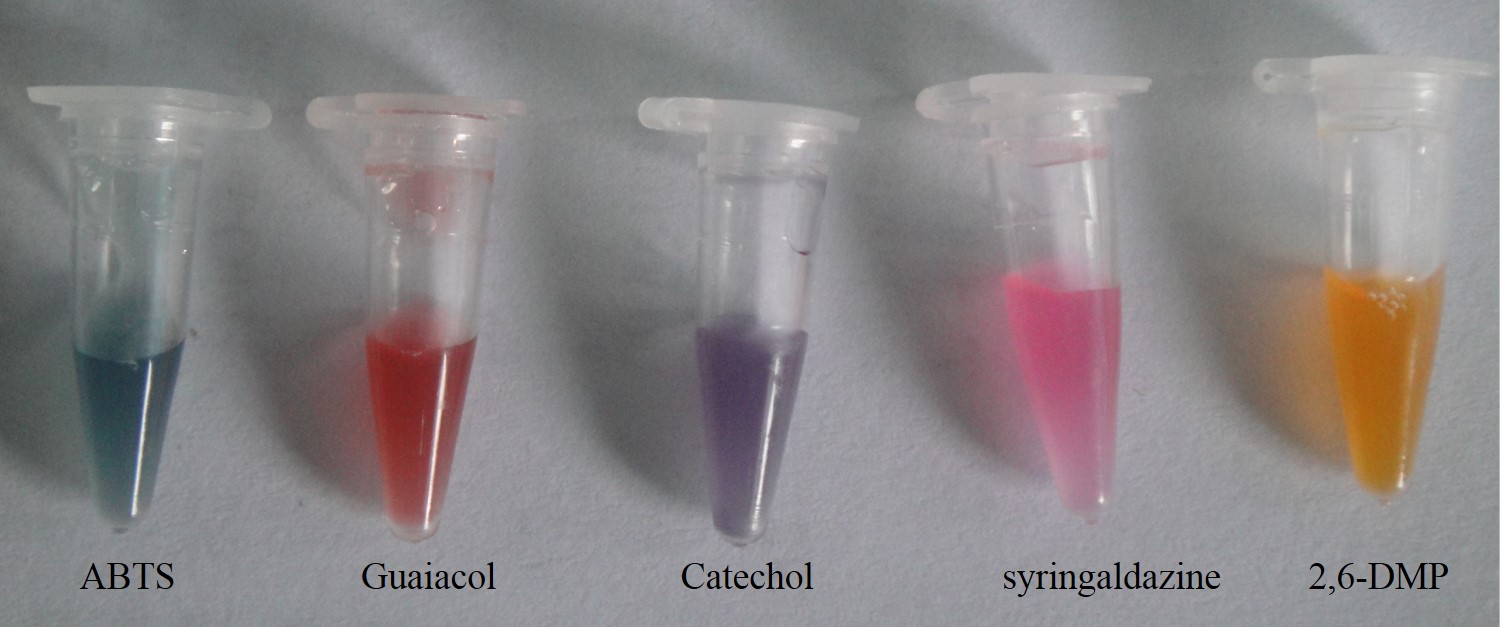

Supplement: FIGURE S4 — The color reaction of the purified recombinant Lac1326 against several substrates. [file Image_4.JPEG]
